# A Probabilistic Three-Dimensional Finite Element Model of a Cemented Hip Implant Failure Under Aseptic Loosening: A Case-Based Probabilistic Framework

**DOI:** 10.3390/bioengineering13060623

**Published:** 2026-05-27

**Authors:** Daniel Truong, Scott J. Hazelwood, Jonathan Fow, Lanny V. Griffin

**Affiliations:** 1 Biomedical Engineering Department, California Polytechnic State University, San Luis Obispo, CA 93407, USA; dmj96744@gmail.com (D.T.); shazelwo@calpoly.edu (S.J.H.); 2 Pacific Central Coast Health Centers, Dignity Health, 850 Fair Oaks Ave Ste 100, Arroyo Grande, CA 93420, USA; jfow@aol.com

**Keywords:** finite element modeling, fracture, fatigue, total hip arthroplasty, probabilistic, Monte Carlo simulation

## Abstract

**Background:** Hip implant fractures are rare, yet difficult to correct once they occur. For cemented implants, fracture is often associated with increased stresses at the implant stem when proximal regions of the implant have debonded. While deterministic analyses offer predictive power by using averages for model inputs, averages fail to capture the variability inherent in device manufacturing and musculoskeletal biology. This study developed a probabilistic finite element model of a debonded hip implant to better account for some of these variabilities to predict the most likely failure mode. The hypothesis was that fatigue would be more likely to occur than overloading. **Methods and Materials**: Monte Carlo sampling generated 1000 simulations varying the material elastic modulus (implant, cement, and bone) and loading magnitude at stance phase of the gait. The resultant distributions of maximum von Mises stress at the stem were compared to distributions for failure properties in the literature. **Results**: The analysis found the likelihood of the implant failing due to overloading was remote. In contrast, fatigue failure had a 99.4% chance of occurring. Fracture mechanics predicted that the debonded implant would reach critical flaw length between 1.8 and 26.4 months, with a mean of 7.2 months. **Conclusions:** The results show good agreement with the findings of the case study the model was based on, particularly in predicting the location of failure and fatigue life. The results of this study provide a framework for developing future decision-making tools that ultimately may assist clinicians in deciding when interventions are necessary to minimize the risk of implant or periprosthetic fracture.

## 1. Introduction

Many aspects of total hip arthroplasty (THA) design are often investigated to ensure success in clinical settings. Computational methods such as finite element analysis allow researchers to vary design parameters related to the THA’s head and cup [1,2,3] or stem [4,5] to reduce stresses. However, such studies may be limited in their ability to predict when an implant may fail in nonideal circumstances, such as when a cemented implant loosens.

Aseptic loosening is often a sufficient condition for revision; however, left untreated, the THA may fail due to fracture [6]. THA fractures require revision surgery to remedy, which generally has lower success rates than the initial implantation [7]. Wear particles from the articulating surfaces of implants (typically polyethylene microparticles) are considered the leading cause of osteolysis in the surrounding bone tissue, leading to aseptic loosening [6]. As bone quality diminishes, a greater fraction of the load experienced at the hip joint is transferred to the bone cement and implant. Over time, the bone cement is unable to support these loads, leading to debonding from the implant proximally, resulting in elevated stresses at the implant stem, which is a common fracture site [8].

There are many ways to investigate fractures due to aseptic loosening in the literature. The most straightforward method is to investigate the bone cement-implant interface to avoid debonding from occurring. Nuño and Amabili [9] found that residual stresses from cement curing increased stresses at the bone cement-implant interface. Mann and coworkers [10] reported frictional interactions at the interface. While both studies investigated factors that contributed to debonding, Nicolella et al. [11] provided a prediction for the likelihood that a cemented implant will be debonded via cement mantle fatigue or shear stress at the interface using probabilistic methods. The results of Nicolella et al.’s study suggest that debonding occurs 40.8% to 44.8% of the time, depending on the failure mode.

Even when a THA has debonded, the implant may still be usable for some time afterward. Nevertheless, when the implant fails, analysis of the failures shows signs of fatigue [12,13]. ISO 7206-4 is an industry standard for predicting fatigue life for implants experiencing fatigue [14]. There are several studies that use ISO 7206-4 as the basis for their prediction of THA durability [15,16,17]. However, there are several drawbacks to this approach. One study found that ISO 7206-4 may be a lower bound for the possible loads an implant may experience in vivo and therefore may not be conservative [18].

Senalp et al. [19] remedy this problem in their study by discretizing loads and including muscle forces rather than depending on a singular lumped force. Despite the strengths of this inclusion, Senalp et al.’s finite element model, like many of the studies discussed thus far, was deterministic. Deterministic studies assume average values for inputs to the model. While averages typically are acceptable representations of a population, they fail to capture population variability. Properties such as bone stiffness can vary greatly between individuals. Thus, relying on averages to predict the outcome of an individual case may lead to over- or underestimating key outputs, such as stress or strain.

One solution to address the limitations of a deterministic approach is to use a probabilistic approach. Rather than relying on a single value to represent the population (sometimes a minimum value), inputs are represented as distributions to capture inherent variability of the data. Thus, the results are distributions of values as well. There are several methods for sampling values to form the inputs, but Monte Carlo simulations are considered the gold standard [20]. Monte Carlo simulations are guaranteed to converge to the true output distribution. While they are among the most computationally expensive sampling methods, requiring significantly more inputs compared to other sampling methods like Most Probable Point [20] or Response Surface methods [21], modern computational tools are significantly faster than in the past, and thus, computational time is less of an issue.

The purpose of this study was to create a probabilistic finite element model for a distally bonded THA to evaluate the most likely failure mode of a cemented hip stem. The model for this investigation was based on one case of an isolated hip stem fracture of a cemented femoral stem without a periprosthetic fracture. The hypothesis for this investigation was that fatigue would be more likely to occur than mechanical overloading based on results from studies in the literature [5,15,17]. As a mechanistic and probabilistic framework, this methodology may enable development of patient-specific tools capable of enabling data-driven interventions by clinicians.

## 2. Materials and Methods

**Figure 1:**
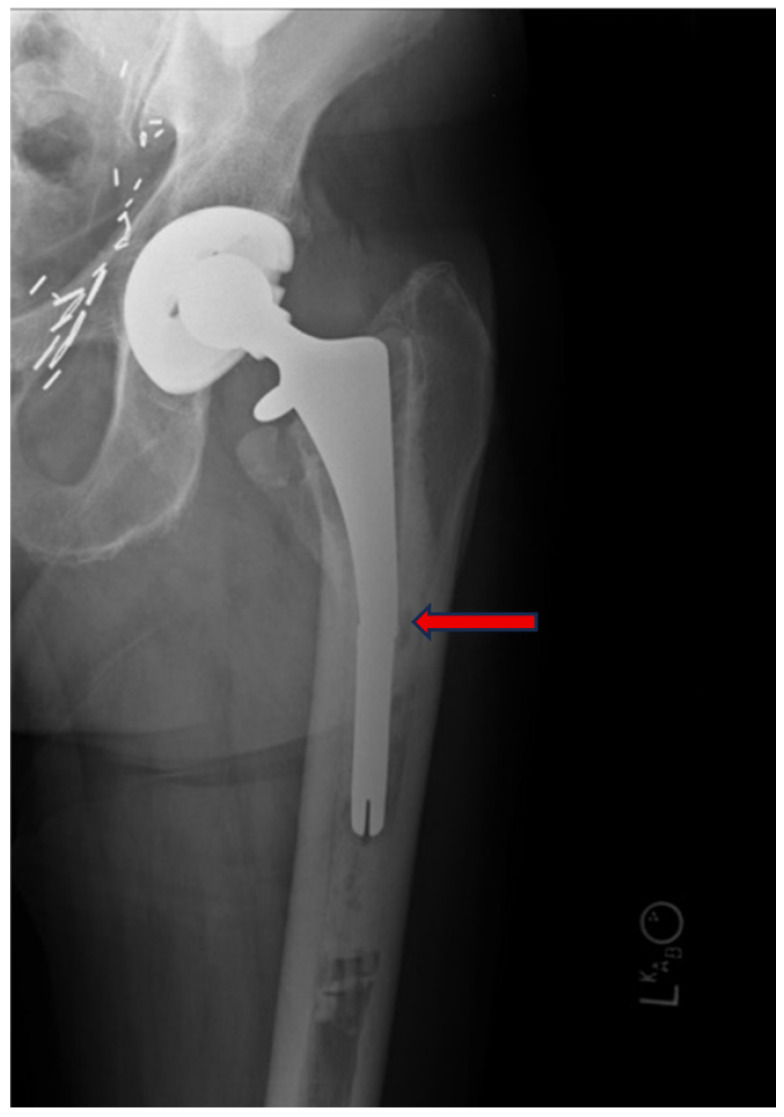
X-ray of the hip implant fracture location, denoted by the red arrow.

This study is a case report of a failed hip stem retrieved from an 83-year-old male (87 kg) patient. The implant was a collared Richards cemented femoral stem with an alumina femoral head and was in use for 14 years before requiring revision due to implant fracture. Routine follow-up radiographs indicated that aseptic loosening had occurred. Six months later, the implant fractured about 65 mm from the distal end (Figure 1). The acetabular cup showed significant wear, as the collar impinged on the cup, which was the primary cause of wear debris that led to osteolysis. The California Polytechnic State University Institutional Review Board determined that this research does not meet the definition of human subjects research, as the study involved a de-identified orthopedic implant retrieved from a deceased individual with no interaction with living human participants or access to identifiable information (determination letter dated 15 May 2026).

**Figure 2:**
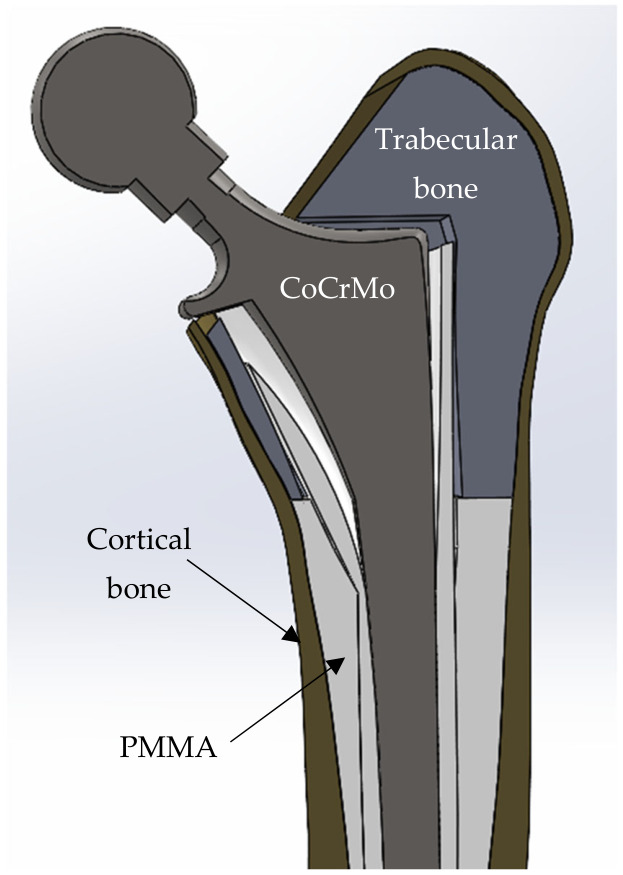
Cross-sectional view of the computer-generated debonded implant model used in this study.

A three-dimensional finite element method (FEM) model of the femur was developed using a computer tomographic (CT) scan of a representative femur from a previous study by Lawrence Livermore National Laboratories (LLNL, Livermore, CA, USA). The FEM model of the implant was developed in Solidworks (v2021, Dassault Systèmes, Vélizy-Villacoublay, France) using dimensions from the fractured implant. ImageJ (v1.53. National Institutes of Health, Bethesda, MD, USA) was used to measure the gap in the debonded region, which was estimated as 2 mm at its widest point, and the cancellous bone in the X-ray image of the implanted femur of the case study (Figure 1). Solidworks was used to generate the geometry for the cancellous bone, bone cement, and implant. The bone cement mantle was split into 2 regions: an inner bone cement mantle and an outer bone cement mantle. The inner mantle ensured that the distal stem had enough cement for fixation while also capturing the cement debonding at the proximal region. The outer mantle outlined the irregular faces of the inner femur. The FEM model consisted of five separate parts (Figure 2).

**Figure 3:**
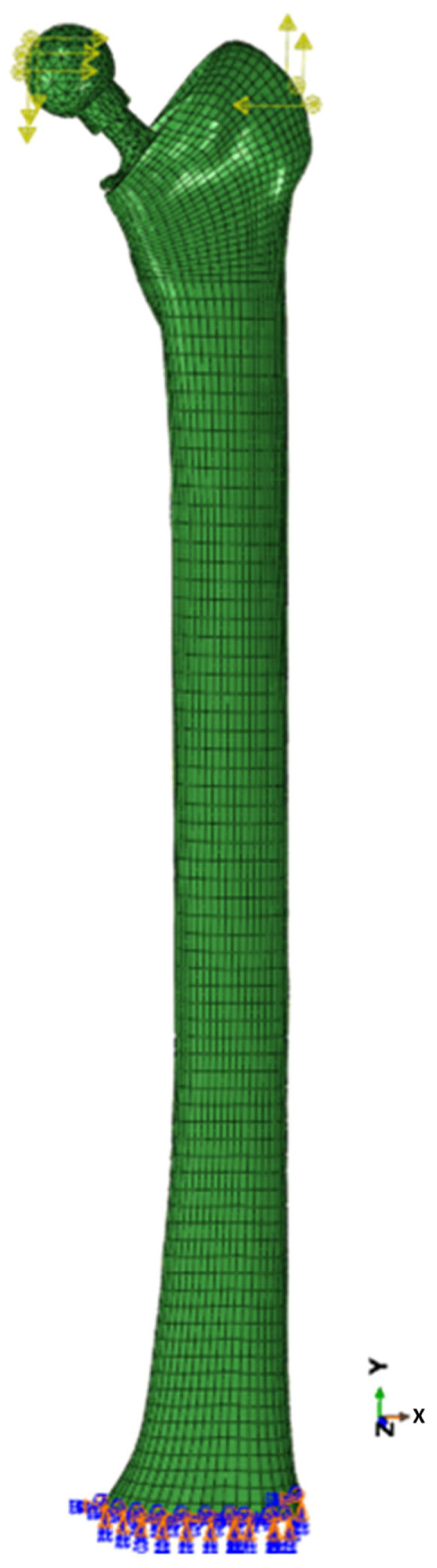
AP view of the meshed femur with loads (yellow arrows) and fixed boundary conditions (red/blue markers).

Once the components were created and set to a position and orientation commonly observed in clinical settings and consistent with the X-ray images available (Figure 1), the parts, except the implant, were meshed using TrueGrid (v4.0.2, XYZ Scientific Applications, Inc., Pleasant Hill, CA, USA). The femur and inner bone cement mantle consisted of quadratic brick elements. The outer bone cement mantle and cancellous bone consisted of linear brick elements. The implant was meshed using quadratic tetrahedral elements in Abaqus’ native mesh generator (Figure 3). The interface between the implant and bone cement in the distally bonded region was modeled using a surface-to-surface tie constraint in Abaqus. The proximally debonded region was modeled using a surface-to-surface contact model with the implant surface denoted as primary and the bone cement as secondary to avoid interpenetration of parts if contact occurred.

The FEA models for the intact femur, the implant, and the implant-femur were validated using published strain data [22] and predicted model principal strains at consistent locations. A FEA model of the ISO 7206-4 configuration was developed to estimate the maximum von Mises stress compared to the distributions of endurance limit and ultimate tensile strength using a Z-test. The final mesh for the implant consisted of 8403 10-node quadratic tetrahedral elements (C3D10R). A mesh convergence study using predicted stress or strain was conducted on all parts prior to the probabilistic analysis to ensure the model was appropriately meshed and converged to a consistent solution (Figure S1).

Abaqus (v6.20, Dassault Systèmes, Vélizy-Villacoublay, France) was used to obtain a static finite element solution. All materials were assumed to be linearly elastic and isotropic. Poisson’s ratio for all materials was assumed to be 0.3. All components, including the bone cement-implant interface, were modeled with tie constraints except where previously noted. For boundary conditions, the distal end of the femur, 43 mm above the femoral condyles, was assumed to be fixed while the hip joint force and lumped abductor force were distributed on the femoral head and greater trochanter, respectively [23]. Table 1 summarizes the random variables in this study, which include mechanical properties for all the components of the model and magnitudes for the components of the abductor force. The hip joint force was made dependent on the abductor force and body weight force to maintain static equilibrium of the femur across the possible force magnitudes sampled.

Monte Carlo sampling was conducted using SIPMath (v3.1, Probability Management, Inc., Palo Alto, CA, USA). Abaqus scripting automated the processing of 1000 cases. Maximum von Mises stress was sampled as the outcome measure along the stem for each model. The probability of failure was determined for two modes: overloading and fatigue. Distributions were created for the possible ultimate tensile strength and endurance limit of CoCr alloy using SIPMath (Table 2); these distributions were evaluated against the distribution of maximum stress.

A sensitivity analysis was conducted with all the random variables: the directional components of the abductor force (Abx, Aby, Abz) and the elastic moduli for the CoCrMo alloy, PMMA, trabecular bone, and cortical bone. Random variables were evaluated at the 10th and 90th percentiles. Maximum von Mises stresses at each percentile were compared to a model defined by the average for each variable.

The fatigue life, *N_f_*, was estimated by an integrated form of the Paris Power Law (Equation (1)), which predicts the number of cycles a flaw requires to reach critical length to failure.

(1) $$N_{f}=\left[\frac{2}{\left(p-2\right)C\gamma^{p}\left(\Delta \sigma \right.{)}^{p}\pi^{\frac{p}{2}}}\right]\left[\frac{1}{a_{i}^{\frac{p-2}{2}}}-\frac{1}{a_{c}^{\frac{p-2}{2}}}\right]$$

In Equation (1), the variables include the Paris law exponent (*p*), the fatigue crack propagation constant (C) (*p* = 8.7 and *C* = 4.0 × 10^−20^ for cast cobalt chrome) [24], and the geometry constant (*γ* = 1.12) [25]. The cyclic stress range (Δ*σ*) was assumed to be equivalent to the maximum value of the von Mises stress in the area of interest, as it was assumed that the minimum cyclic stress was zero during the swing phase (R = 0). The remaining variables are the initial flaw length (*a_i_*) and the calculated critical crack length (*a_c_*). The initial flaw size was based on the typical grain size for CoCrMo [8]. To convert number of cycles into a measure of time, a loading frequency based on average steps per day for older adults with disabilities of 4500 ± 333 was used [26].

Equations (2)–(6) describe the plane strain fracture toughness of a material (*K_IC_*) given the geometry factor (*F*), stress (Δ*σ*), and flaw length (*a*).

(2) $$K_{IC}=F\sigma \sqrt{\pi a}$$

(3) $$F=G\left[0.923+0.199Y^{4}\right]$$

(4) $$G=0.92\left(\frac{2}{\pi }\right)\sec\beta \sqrt{\frac{\tan\beta }{\beta }}$$

(5) Y=1−sinβ

(6) $$\beta =\frac{\pi }{2}\frac{a}{D}$$

The formulation of the geometry factor for a surface crack in a solid cylinder (Equations (3)–(6)) [27] is dependent on the flaw length and the diameter of the cylinder (*D* = 9 mm for the implant stem). The critical flaw length was determined probabilistically using Equation (2) by setting *K_IC_* and Δ*σ* as random variables to solve for *a_c_*. The values for variables used in Equations (2)–(6) are given in Table 2.

## 3. Results

**Figure 4:**
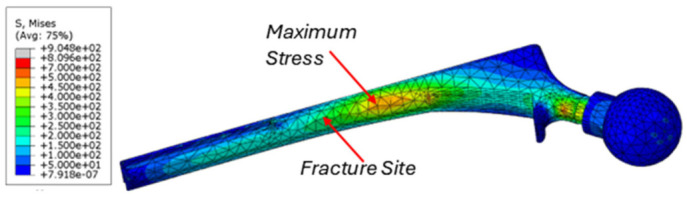
Contour plot of the maximum von Mises stress for the implant. The locations of maximum stress and the actual fracture site are indicated.

The probabilistic FEM model predicted that the maximum von Mises stress occurred 87 mm from the stem base, 32% higher than the actual fracture site observed in the case study (Figure 4). The average maximum stress was 474.2 ± 20.6 MPa.

**Figure 5:**
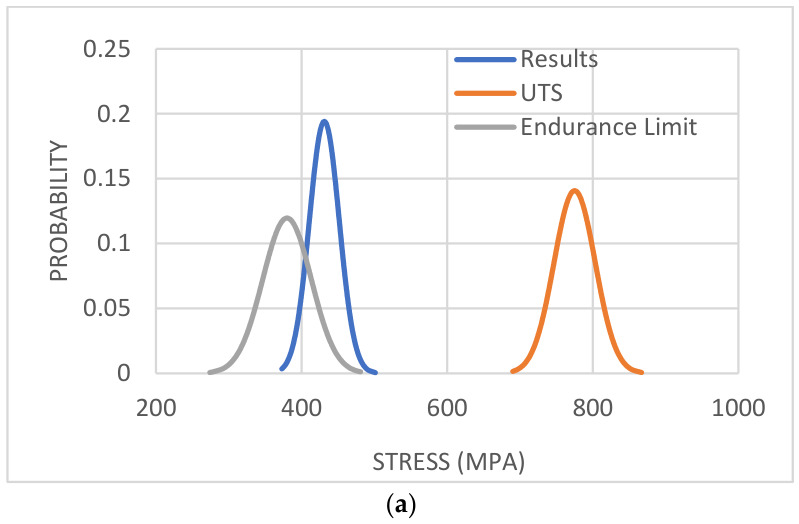

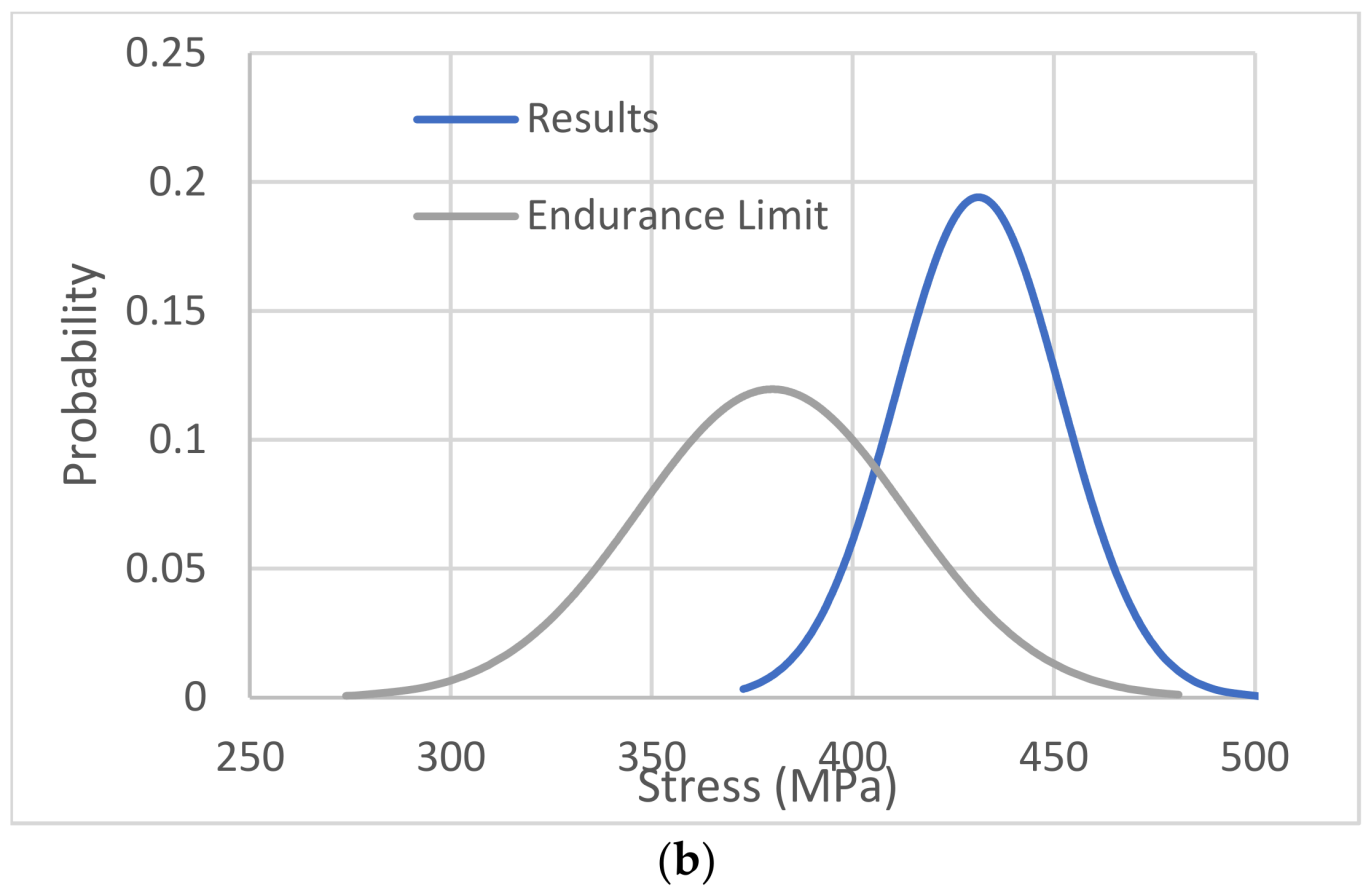
(**a**) Probability density plots of maximum von Mises stress for the implant stem from the FEM model results compared to the endurance limit and ultimate tensile strength (UTS) of CoCrMo. (**b**) FEM model results compared to the endurance limit.

Figure 5 shows the distribution of the resultant stresses in the implant and the failure properties of CoCrMo. The probability of failure due to implant stress exceeding the ultimate tensile strength (overload) was virtually 0% since the implant stress has no apparent overlap with the UTS. However, the probability of failure resulting from fatigue, as indicated in the overlap of the endurance limit and FEM model probability distributions in Figure 5, was 99.4%.

A FEA model of the ISO 7206-4 for the Richards cemented femoral stem estimated that the maximum von Mises stress in the stem was 660 MPa. The Z-test found that the ISO 7206-4 model predicts failure by fatigue (*p* > 0.9999) but not by overload (*p* = 2.0 × 10^−5^).

**Figure 6:**
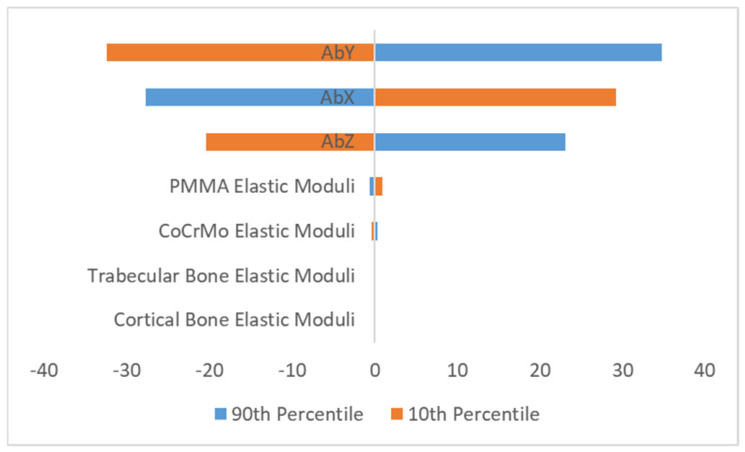
Sensitivity analysis of the properties of the random variables in the model.

**Figure 7:**
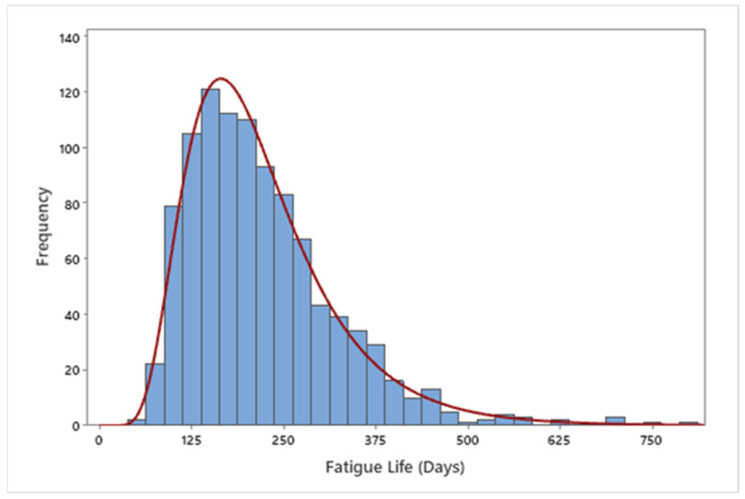
Predicted implant fatigue fit to a lognormal distribution (red line).

The sensitivity analysis found that abductor forces generally had a greater effect on the maximum stress variation than material properties (Figure 6). The proximal-distal component of the abductor force (Ab_Y_) had the greatest effect of all force variables tested. Fatigue life varied between 55 and 803 days. Average fatigue life was 220.4 days ± 102.0 days (Figure 7).

## 4. Discussion

**Figure 8:**
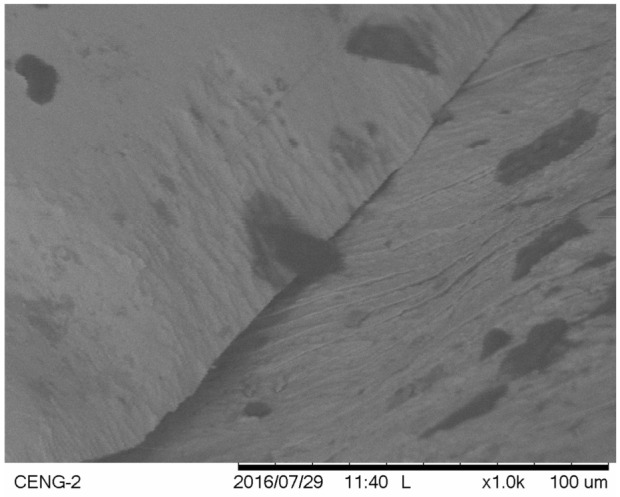
Scanning electron micrograph of the implant fracture site showing signs of fatigue.

The purpose of this study was to develop a probabilistic finite element model for a distally bonded THA to evaluate the likelihood of the implant failure mode. The hypothesis was that fatigue would be more likely to occur than overloading. The average maximum von Mises stress (474.2 MPa) was well below the UTS of CoCrMo, but the average stress was above CoCrMo’s endurance limit, which suggests fatigue would be more likely to occur (Figure 5). Probabilistically, overloading was not likely, but fatigue was practically certain. Returning to the case study, scanning electron microscopy in the vicinity of the fracture site revealed striations indicative of fatigue, further supporting the conclusion that fatigue was prevalent (Figure 8).

The model prediction of the fracture site location was 22 mm proximal to what was observed. This discrepancy is likely due to simplifying assumptions, such as using a generic femur model, a simplified debonded region, and modeling where the debond ended. The specific location of the debond was estimated from the X-ray (Figure 1). The model location of maximum stress was close to this site, which is about 20 mm proximal, and the von Mises stress is relatively uniform in this range (Figure 4).

The model’s utility is further demonstrated when considering fatigue life. Applying fracture mechanics to the maximum stress results predicted that the implant stem would most likely fracture unstably after 7.2 ± 3.4 months. The results best fit a lognormal distribution (Figure 7), which is consistent with empirical observations [28]. The implant was in use for 14 years before requiring replacement. Radiographs for this case study documenting implant debonding to fracture were taken 6 months apart from each other. Thus, this model was able to provide an appropriate prediction based on the time that debonding was first detected.

The loading frequency used for this study was based on populations of older individuals with disabilities [26]. Considering that the subject was in his early 80s and had a hip replacement, the assumption of walking one or two miles seems justified. If the subject was more active, the time to failure would be shortened.

The sensitivity study found that abductor loads had a greater effect on the maximum stress experienced by the implant stem compared to material properties of either the cement or bone. Dopico-Gonzalez et al. probabilistically investigated bone strain in a properly functioning uncemented implant [29]. They found the results were most sensitive to joint loads and the elastic modulus of the bone. However, their study was very different than this study in that they were evaluating strain in the bone with a fully bonded cementless implant, presumably with a view toward understanding stress shielding effects. Furthermore, their study used a much more simplified loading case. In that case, it would seem to be reasonable that bone stiffness affects strain transfer.

Nicolella et al. [11,21] investigated cemented THA using probabilistic methods. They were primarily interested in the failure of the implant-to-cement interface, but that study did not present a sensitivity analysis [11]. The second study found that the cement/implant interface failure was not sensitive to cement or bone mechanical properties, but very sensitive to joint loads and interfacial shear strength [21]. Eastly et al. reported that the results were also not sensitive to bone elastic properties in a parametric study performed in accordance with ISO 7206-4 [30]. Therefore, the results of our sensitivity analysis appear to be consistent with those found in the literature.

One explanation for this outcome is that debonding caused most of the load to transfer to the implant stem rather than the surrounding bone and bone cement. This is supported by the stress contour plot of the implant (Figure 4). This would explain why material properties for the two bone types and bone cement had little effect on the results. The minimal effect of implant material properties on maximum stress may be due to how the mechanical properties of CoCrMo varied from the mean value. While the loads had a standard deviation of 10% of the mean, the CoCrMo elastic modulus standard deviation was 3% of its mean since the elastic modulus for this alloy ranges between 210 and 250 GPa.

The probabilistic study can account for property variations when considering unknown material composition. If chemical analysis revealed that traces of boron were present in the CoCrMo, the average endurance limit would increase from 385 to 524 MPa [31]. A deterministic analysis would fail to predict fracture due to fatigue in this case. However, the probabilistic analysis would suggest the probability of failure due to fatigue to be 8.73%. This example demonstrates the utility of probabilistic analyses to quantify risk under uncertain conditions. Even when the composition of CoCrMo is not known, the probabilistic model consistently predicted that the implant would fail despite an increase in the endurance limit. Another potential concern is corrosion fatigue, which could shorten the life of the alloy; however, CoCrMo possesses excellent corrosion resistance even in chloride-rich environments [32].

Most studies concerned with fatigue performance rely on ISO 7206-4, which simplifies the loading schema for in vitro testing but may not accurately replicate the loads an implant may experience [33]. This study considers a three-dimensional probabilistic model that considers in vivo biomechanics at the hip joint at the stance phase of the gait. This approach allows the model prediction to better translate to clinical settings while also remedying the fault inherent when relying on one example to represent a highly varied population. Given the limitations of in vivo hip joint measurements [34], the probabilistic approach is better able to account for uncertain conditions compared to a deterministic analysis. Differences in implant geometry [35], femur geometry [36], and hip biomechanics [37] limit the ability to translate results across other investigations.

## 5. Limitations

One limitation of this study is that it is based on one case. Implant fracture is a relatively rare occurrence compared to periprosthetic fracture, so access to more cases is limited. However, the purpose of this study was primarily to provide a methodical framework to evaluate and ultimately predict failures that may lead to revision so that the clinician can make judgments about when to intervene before catastrophic failure occurs. Therefore, this study has demonstrated promising value in developing tools that may assist implant designers and clinicians.

Patient CT data were not available to develop a personalized FEA model. However, the primary purpose of this study was to evaluate the probability of implant failure due to fatigue. Assuming the geometry was reasonably close, and considering the construct was cemented, the loads applied to the implant should be adequate for the purpose of this study. Furthermore, a validation study was performed using the loads and boundary conditions from an experimental study in the literature, which used patient-specific CT data, with the results being comparable [22], thus indicating that the results of this study are reasonable.

The material properties of bone were modeled assuming isotropic elasticity, which is, strictly speaking, not valid. However, using a patient-specific model developed using CT data would still be modeled by linear isotropic elasticity, but the material properties for the elements would be based on the Hounsfield number, which correlates to density and elastic modulus [38]. Poisson’s ratio value for PMMA used for this study was 0.3, which is lower than reported values. However, since this study is concerned with a fully debonded implant, there should be no shear stresses developed in the region of interest, and therefore, the choice of Poisson’s ratio should not greatly influence the conclusions of this study. Furthermore, trying to model anisotropic effects is challenging and perhaps unfeasible since the directions of the material principal axes do not coincide with the standard SAE coordinate system typically used in the modeling of humans and are not attainable from CT data. Riesinger et al. experimentally determined that the mean helix angle of anisotropy was 10.3° with respect to the AP axis of long bones, and that the degree of anisotropy was 1.75 [39]. Furthermore, similar studies have demonstrated that elastic modulus has a negligible effect on implant failure [11,21]; therefore, it seems likely that interactions involving elastic modulus would similarly have a negligible effect.

While the distributions of flaw size and trabecular bone modulus may be skewed, the coefficient of variation is small enough that negligible probability mass falls below zero. For this study, none of the statistical quantities fell into physically impossible ranges, such as negative moduli or flaw sizes. Using the Paris law without knowledge of the initiation region is a limitation. However, assuming a pre-existing initial flaw size of about 0.2 microns is reasonable. Using the calculated von Mises stress when the implant is debonded results in a Δ*K* greater than the crack initiation threshold, and so crack growth is predicted to occur. The fatigue life was then estimated as the time to grow to a critical length.

Assuming R = 0 is likely to be conservative, as having additional compressive stresses due to pre-tension or residual stress would tend to lower the stress range in the compressively loaded regions. In regions of tensile stress, the compressive stresses would contribute to crack closure.

Using the stance phase of walking instead of more realistic cases to include going up or down stairs, or including slips, trips, and falls, is not necessarily conservative, but reflects a reasonable first approximation. Since fatigue is still predicted, and there is SEM evidence of fatigue (Figure 8), the model predictions appear reasonable.

Despite these limitations, this probabilistic finite element model is an advancement over previously published total hip arthroplasty models in several ways. This model offers a more realistic prediction of in vivo fatigue performance for cemented hip implants by replicating implantation anatomy and hip biomechanics rather than relying on simplified assumptions based on ISO 7206-4. It also considers inherent uncertainty in modeling properties and loads to provide a more holistic prediction. Thus, this model considers a limited distribution of possible inputs to produce a distribution of failure rather than output a deterministic answer that may be based on faulty assumptions.

Measurement of hip implant performance in vivo is limited [34], and in vitro results often do not translate to clinical settings. With the methodology described, this modelling technique is a cost-effective tool for designers to create improved hip implants. With further refinement, this model may be adapted to enable clinicians to determine when clinical interventions may be warranted. These may be detected by providing a quantitative risk assessment associated with radiographic evidence of osteolysis, thigh pain, or bone density scans.

While this study only evaluated implant fracture, periprosthetic fracture accounts for about 15% of hip revisions and about 20.5% of early hip revisions [40]. Furthermore, while cementing implants is not as common in the US as compared to other countries, Tanaka and coworkers reported that cemented fixation lowers the risk of complications, including periprosthetic fracture, compared to cementless fixation [41]. Therefore, cementing may ultimately become more common in patients who are susceptible to periprosthetic fracture. Follow-on studies could examine the effects of bone density, materials, fixation type, loads, and implant design contributing to periprosthetic fractures, femoral neck fractures, or other complications.

## 6. Conclusions

The strengths of this study and other similar ones may enhance clinical decision-making by accounting for natural variation of parameters. This study used a 3D Monte Carlo FEA model and concluded that fatigue was the most likely cause of implant failure. Further work is needed to evaluate the risk of periprosthetic fracture, which would be better suited to patient-specific models.

## Figures and Tables

**Table 1:** Properties and forces used for the probabilistic model.

Variable	Mean ± SD	Range (Min, Max)	Distribution
Cortical Bone Modulus (GPa)	14.00 ± 0.75	11.7, 16.4	Normal
Trabecular Bone Modulus (GPa)	1.75 ± 0.50	0.14, 3.3	Normal
CoCrMo Modulus (GPa)	220 ± 6.67	200.0, 241.6	Normal
PMMA Modulus (GPa)	1.90 ± 0.15	1.5, 2.4	Normal
Abductor Force, X (N)	526 ± 53	364.4, 697.3	Normal
Abductor Force, Y (N)	838 ± 84	586.6, 1110.4	Normal
Abductor Force, Z (N)	334 ± 34	240.4, 455.1	Normal

**Table 2:** Distributions used to model CoCrMo failure.

Variable	Mean ± SD	Range (Min, Max)	Distribution
Ultimate Tensile Strength (MPa)	775.0 ± 28.3	689.9, 866.9	Normal
Endurance Limit (MPa)	383.5 ± 30.3	273.9, 481.8	Normal
Initial Flaw Size (mm)	0.19 ± 0.011	0.15, 0.23	Normal
Fracture Toughness (MPa∙m^0.5^)	65.0 ± 1.66	60.0, 69.4	Normal
Loading Frequency (steps/day)	4500 ± 333	3434, 5543	Normal

## Data Availability

The original contributions presented in this study are included in the article. Further inquiries can be directed to the corresponding author.

## Supplementary Materials

The following Supporting Information can be downloaded at: https://www.mdpi.com/article/10.3390/bioengineering13060623/s1, Figure S1: A mesh convergence study for the implant.

## Abbreviations

The following abbreviations are used in this manuscript:

NIH	National Institutes of Health
ISO	International Organization for Standardization
PMMA	Poly(methyl-methacrylate)
CoCrMo	Cobalt-chrome-molybdenum alloy
Ab	Abductor
*K_IC_*	Mode 1 plane strain fracture toughness
UTS	Ultimate tensile strength
THA	Total hip arthroplasty
FEM	Finite Element Method
CT	Computed tomography
SEM	Scanning electron microscopy
AP	Anterior-posterior
SAE	Society of Automotive Engineers
