# Supplementary figures and images for: A Probabilistic Three-Dimensional Finite Element Model of a Cemented Hip Implant Failure Under Aseptic Loosening: A Case-Based Probabilistic Framework
Source: Bioengineering (Basel). 2026 May 27;13(6):623. doi: 10.3390/bioengineering13060623 (PMC13296152; doi:10.3390/bioengineering13060623)

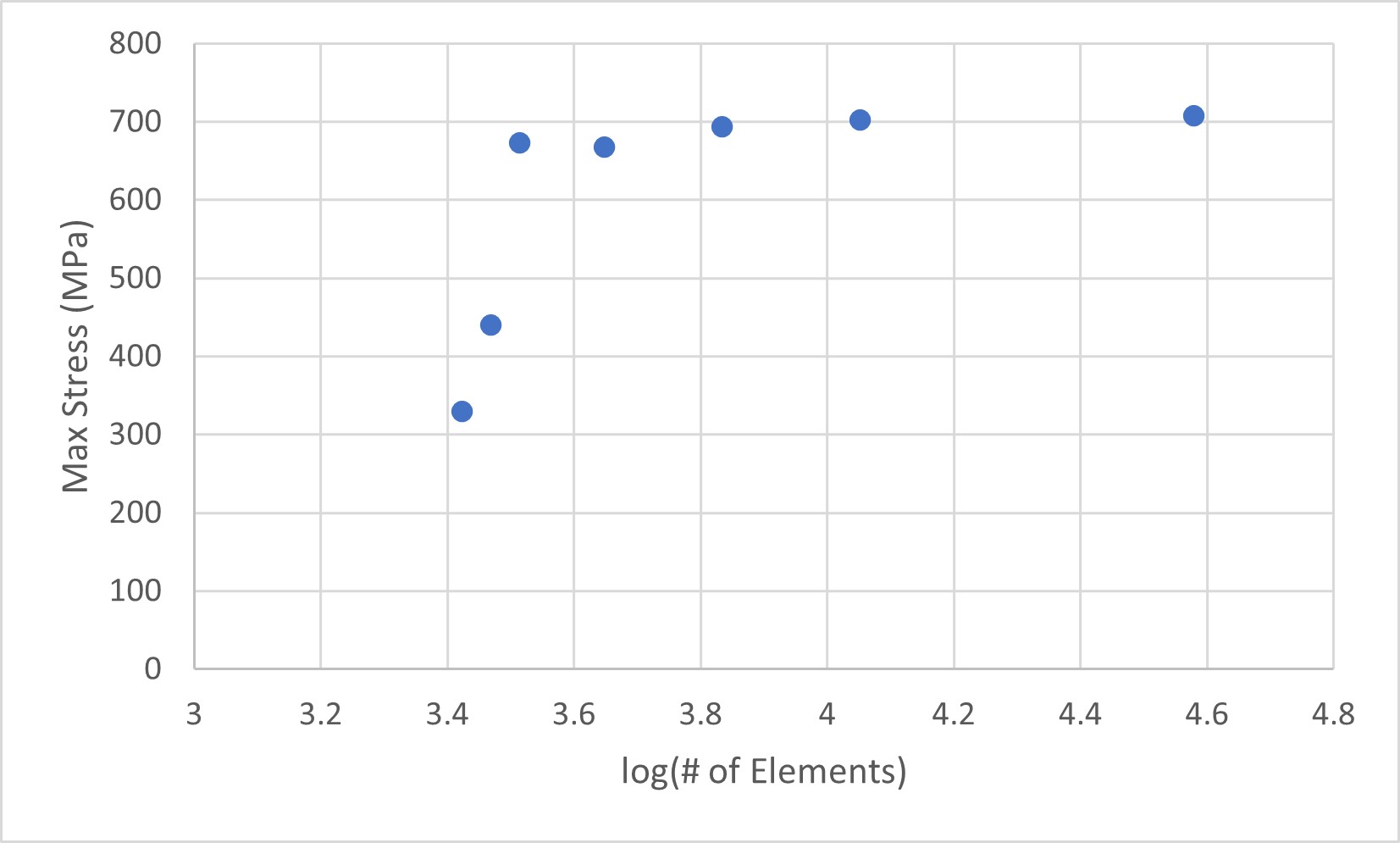

Supplement: Supplementary file 1 [file bioengineering-13-00623-s001.zip › bioengineering-4294331-supplementary.tif]
